# 49 Bromelain Based Enzymatic Debridement of Pediatric Deep Burns: Top Line Results of a Multicenter RCT

**DOI:** 10.1093/jbcr/irac012.052

**Published:** 2022-03-23

**Authors:** Yaron Shoham, Ravi P Narayan, Stan Monstrey, Henk Hoeksema, Giavonni M Lewis, Manoj Kumar Jha, Shawn D Larson, Oleksii Kravtsov, Elena M Hanganu, Horea G Gozar, Tam N Pham, Adam Singer, Robert L Sheridan, Paul M Glat, Frank Sander, Dhaval Bhavsar, Lucy Wibbenmeyer

**Affiliations:** Soroka University Medical Center, Beer Sheba, HaDarom; ESIC Medical College & Hospital, Faridabad, New Delhi, Delhi; Ghent University Hospital, GENT, Oost-Vlaanderen; University Hospital Ghent, Gent, Oost-Vlaanderen; University of Utah Burn Center, Salt Lake City, Utah; ABVIMS & Dr. RML Hospital, New Delhi, Delhi, Delhi; University of Florida College of Medicine, Gainesville, Florida; State Institution «Zaitsev VT Institute of General and Emergency Surgery of the National Academy of Medical Sciences of Ukraine», Kharkiv, Kharkivs'ka Oblast'; University of Medicine and Pharmacy, Faculty of Medical Bioengineering, Balneophysiotherapy and rehabilitation, Iași, Iasi; University of Medicine, Pharmacy, Science and Technology of Târgu Mureș, Targu Mures, Mures; University of Washington, Seattle, Washington; Stony Brook University Medical Center, Stony Brook, New York; Shriners Hospitals for Children Boston, Boston, Massachusetts; St Christopher's Hospital for Children, Bala Cynwyd, Pennsylvania; Center for Severe Burns with Plastic Surgery, Berlin, Berlin; University of Kansas Health System, Kansas City, Kansas; University of Iowa MD, FACS, Iowa City, Iowa

## Abstract

**Introduction:**

Bromelain based debridement (BBD) of deep burns with a concentrate of proteolytic enzymes enriched in Bromelain is approved for use in adults in several regions worldwide. Children are a large part of the patient population in many burn centers around the world. Clinical trial experience and off label reports point to BBD safety and efficacy in children as well. The aim of this study was to further assess the safety and efficacy of BBD in children, in efforts to support regulatory approval for the use of BBD in children.

**Methods:**

One hundred and forty five children aged 0-18 years old suffering from deep thermal burns between 1-30% TBSA were enrolled in a multicenter, multinational, open label, randomized, controlled phase III study. Seventy two children were randomized to eschar removal with BBD and 73 children to standard of care (SOC) surgical and/or non-surgical eschar removal methods, at the investigators' discretion. Patients who did not achieve complete eschar removal after BBD application were rescued with SOC eschar removal methods. Wound care after achieving complete eschar removal was according to routine methods, at the investigators' discretion. Patients are currently in stages of long term follow-up, planned for a duration of >2 years. This abstract reports the top line results of the study including the first year of follow-up.

**Results:**

Baseline characteristics were similar between the arms. The median age was 3.4 years in the BBD arm and 3.9 years in the SOC arm. The average burn area was 7.0±4.9 %TBSA in the BBD arm and 6.2±4.8 %TBSA in the SOC arm. The study met all 3 primary endpoints: Median time to complete eschar removal was 1 day for BBD and 6 days for SOC (p< 0.001), the percent wound area excised in order to complete eschar removal was 1.5% for BBD and 48% for SOC (p< 0.0001), and the MVSS scores at 12 months were 3.83 for BBD and 4.86 for SOC (non-inferiority endpoint). Secondary endpoints demonstrated 8.3% incidence of surgical excision to complete eschar removal for BBD and 64.4% for SOC (p< 0.0001), mean eschar removal associated blood loss of 32±284ml for BBD and 202±409 for SOC (NS), a 25.9% incidence of autografting in deep partial thickness wounds for BBD and 37.7% for SOC (p=0.054), and a mean percent area of deep partial thickness wound autografting of 15.9±38.6 for BBD and 22.8±43.7 for SOC (NS). Safety endpoints demonstrated a non-inferior time to complete wound closure (median 32 days for BBD, 34 days for SOC) and no significant safety issues were demonstrated during the study.

**Conclusions:**

BBD was shown to be a safe and effective debridement agent in pediatric burns.

